# Supplementary material for: Predicting Essential Components of Signal Transduction Networks: A Dynamic Model of Guard Cell Abscisic Acid Signaling
Source: PLoS Biol. 2006 Sep 12;4(10):e312. doi: 10.1371/journal.pbio.0040312 (PMC1564158; doi:10.1371/journal.pbio.0040312)
Supplement: Supplementary file 1 [file pbio.0040312.sg001.pdf]

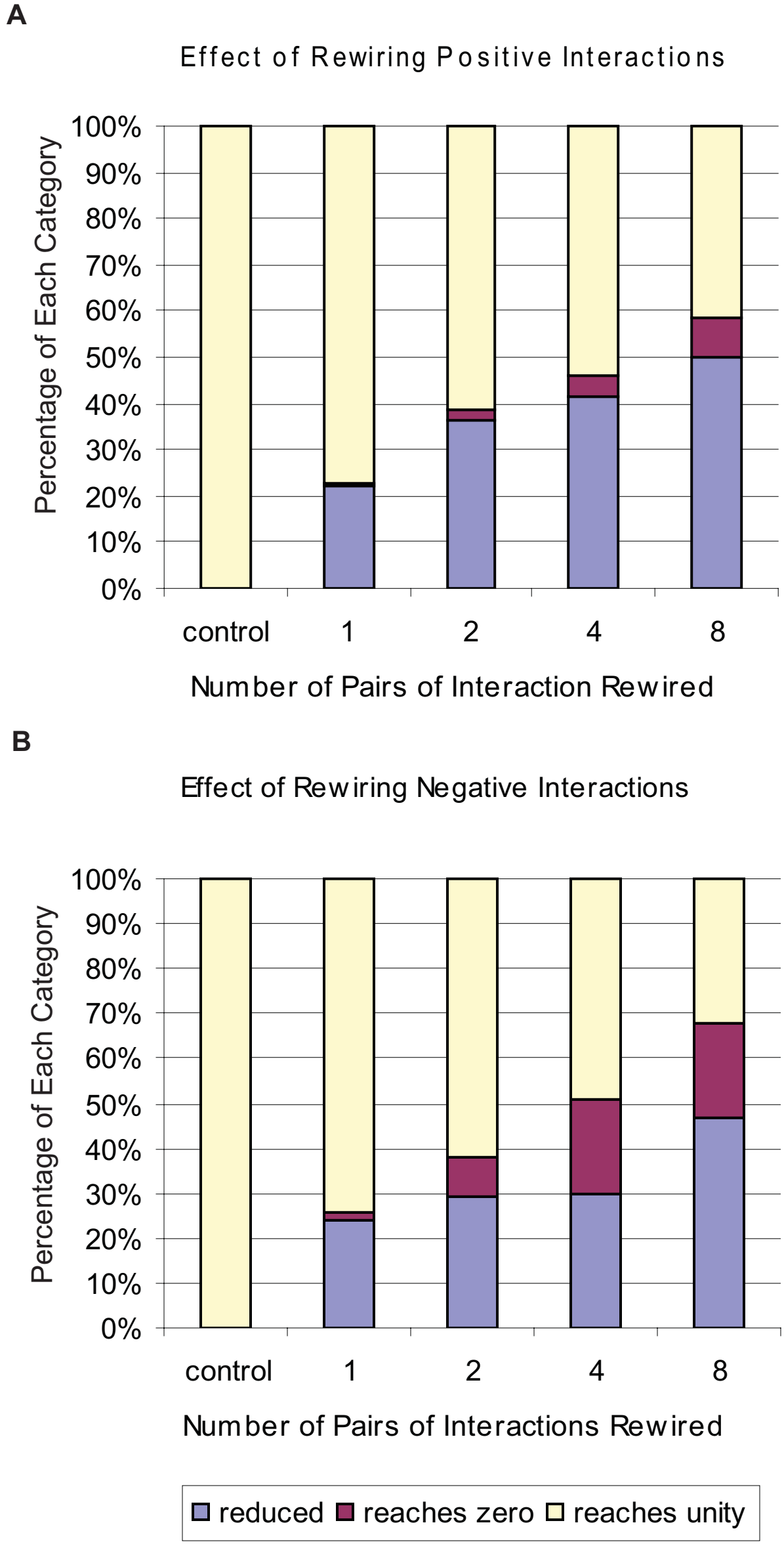
A
Effect of Rewiring Positive Interactions
100%
90%
80%
70%
60%
50%
40%
30%
20%
10%
0%
Percentage of Each Category
control
1
2
4
8
Number of Pairs of Interaction Rewired
B
Effect of Rewiring Negative Interactions
100%
90%
80%
70%
60%
50%
40%
30%
20%
10%
0%
Percentage of Each Category
control
1
2
4
8
Number of Pairs of Interactions Rewired
reduced
reaches zero
reaches unity
