# Supplementary material for: Predicting Essential Components of Signal Transduction Networks: A Dynamic Model of Guard Cell Abscisic Acid Signaling
Source: PLoS Biol. 2006 Sep 12;4(10):e312. doi: 10.1371/journal.pbio.0040312 (PMC1564158; doi:10.1371/journal.pbio.0040312)
Supplement: Supplementary file 2 [file pbio.0040312.sd001.doc]

**Text S1: Detailed Justification for Each Boolean Transfer Function**

**SphK ^*^ = ABA**

**S1P ^*^ = SphK**

**GPA1^*^ = (S1P or not GCR1) and AGB1**

**PLD^*^ = GPA1**

**PA ^*^ = PLD**

ABA-induced phosphatidic acid production is mediated by the S1P pathway and heterotrimeric G proteins [1]. ABA activates SphK, which produce S1P, and heterotrimeric G proteins are downstream of the S1P signal [1]. ABA induced S1P production peaks at 5 minutes after ABA stimulus, then decreases to a level above the resting level [1]. This dynamic behavior implies a negative feedback acting on S1P production. As nothing is known about the putative S1P feedback inhibitor, and there is no evidence that nodes downstream of S1P have transient behavior, we do not explicitly include negative feedback into the rule describing S1P activity. AGB1 is the sole Arabidopsis Gβ subunit [2]. GCR1 is a negative regulator of the ABA and S1P signal in guard cells and it can interact with GPA1 [3]. The *gcr1* mutant shows stronger ABA response than the wild type at moderate ABA concentrations, but interaction with GCR1 does not block G protein signaling, thus we model this negative regulation by an “or not” function. In *Arabidopsis*, the only canonical G protein α subunit, GPA1, can interact with PLDα1 and activates phospholipase D activity [4]. Phospholipase D catalyzes the production of phosphatidic acid (PA). Although there is no direct experimental evidence for S1P-induced PLD activity, the above observations taken together imply that S1P activates PLD through GPA1 and activated PLD catalyzes PA production. We assume that the precursors of SphK, PLD, and the activity of AGB1 are not limiting factors for the signal transduction process.

**pH_c_ ^*^ = ABA**

ABA induces cytosolic pH increase (pH_c_) [5]. It has been shown that pH_c_ increase starts immediately after ABA stimulus [6]; cytosolic pH reaches a peak value at 15 minutes then decreases steadily, stabilizing at a level above the resting level [5]. Since nothing is known about the putative pH_c_ feedback inhibitor, and there is no evidence that nodes downstream of pH_c_ have transient behavior, we do not explicitly include negative feedback into this rule.

**OST1^*^ = ABA**

**ROP2^*^ = PA**

**Atrboh^*^ = pH_c_ and OST1 and ROP2 and not ABI1**

**ROS^*^ = Atrboh**

At least three pathways have been shown to regulate reactive oxygen species (ROS) production (catalyzed by Atrboh) in Arabidopsis. ABA failed to elicit ROS production in an *ost1* mutant [5,7]. Our unpublished data and other indirect evidence also suggest that PA participates in the ABA-induced ROS production pathway [8]. The third pathway is the cytosolic pH pathway; if cytosolic pH is clamped by butyrate, ABA-induced ROS production is also inhibited [5]. ABA-induced ROS production is inhibited in the *abi1-1* dominant mutant [9], we model the combination of activators (pH, OST1, ROP2) and inhibitor (ABI1) as an “and not” function.

**H^+^ ATPase^*^ = not ROS and not pH_c_ and not Ca^2+^_c_**

The H^+^ ATPase has been shown to be inhibited by reactive oxygen species [10], cytosolic calcium concentration increase [11] and cytosolic H^+^ concentration increase [12]. The “and” function reflects the assumption that any of the three negative regulators is sufficient to inhibit the H^+^ ATPase activity.

**ABI1^*^ = pH_c_ and not PA and not ROS**

ABI1 has been shown to be upregulated by cytosolic pH [13], and negatively regulated by ROS [14]. A recent paper shows that ABI1 is relocated from the cytosol to the plasma membrane by binding to phosphatidic acid [15], therefore PA negatively regulates ABI1 activity. In the equation, we use the “and” function to signify that the activation of ABI1 requires an increase in cytosolic pH and either negative regulator’s presence can efficiently reduce the strength of ABI1.

**RCN1^*^= ABA**

**NIA12^*^= RCN1**

**NOS^*^ = Ca^2+^_c_**

**NO^*^ = NIA12 and NOS**

**GC^*^ = NO**

**ADPRc^*^ = NO**

**cADPR^*^ = ADPRc**

**cGMP^*^ = GC**

These seven equations plus the CIS rule given below encapsulate the nitric oxide dependent pathway of induced intracellular Ca^2+^ release. ABA-induced NO production through the NOS pathway is calcium dependent [16], we hypothesize that nitric reductase (NIA12) activity is regulated by PP2A(RCN1) based on the well conserved NIA12 regulation mechanism [17]. Both cADPR and cGMP are downstream of the nitric oxide pathway in regulation of guard cell closure [17,18]. NIA and NOS can both mediate NO production. ADP ribose cyclase (ADPRc) catalyzes cADPR formation and guanyl cyclase (GC) catalyzes cGMP formation.

**PLC^*^ = ABA and Ca^2+^_c_**

**InsP3^*^ = PLC**

**InsPK^*^ = ABA**

**InsP6^*^ = InsPK**

ABA induce both InsP3 and InsP6 production in guard cells through the PLC [19] or InsPK pathway. PLC and InsPK represent, respectively, the known last steps in the catalysis of InsP3 and InsP6 production [20]. PLC-catalyzed InsP3 production requires Ca^2+^ [21].

**CIS^*^ = (cGMP and cADPR) or (InsP3 and InsP6)**

In guard cells, multiple intracellular compartments are able to store Ca^2+^ and release it after stimulation. The “and” function between cGMP and cADPR reflects that these two signaling molecules are dependent and may cause Ca^2+^ release from the same compartment. Both InsP3 and InsP6 can induce Ca^2+^ release [19]. InsP6-caused Ca^2+^ release from intracellular stores is not Ca^2+^ dependent [20], and elicits a different type of Ca^2+^ increase compared to InsP3. However, the InsP3 and InsP6 pathways are biochemically linked [22], so we model their synergy as an “and” function. The “or” function between the cGMP/cADPR and Ins3/Ins6 pathways reflects the assumption that each of these pathways can efficiently induce Ca^2+^ increase inside the cell.

**Ca^2+^ ATPase^*^ = Ca^2+^_c_**

**Ca^2+^_c_ ^*^ = (CaIM or CIS) and (not Ca^2+^ ATPase)**

Cytosolic Ca^2+^ concentration can increase during ABA treatment due to Ca^2+^ influx from outside of the cell and/or Ca^2+^ release from certain cellular compartments. In reality, Ca^2+^ release from intracellular compartments can be mediated by different types of transporters localized on different intracellular membranes [23]; however, current experimental data do not provide enough evidence for a more detailed representation. We subsume the mechanisms that mediate Ca^2+^ efflux from the cytosolic compartment, including Ca^2+^ ATPases and Ca^2+^/H^+^ antiporters, as the node Ca^2+^ ATPase in our model. This rule is the simplest way to incorporate Ca^2+^ oscillations which have been observed in guard cells [19,24,25].

**AnionEM^*^ = ((Ca^2+^_c_ or pH_c_) and not ABI1 ) or (Ca^2+^_c_ and pH_c_)**

Anion efflux through the membrane was shown to be upregulated by cytosolic Ca^2+^ and pH increase [26,27]. Anion efflux current can be further divided into fast anion current (R type) and slow anion current (S type). Our rule only models the slow current that is not strongly regulated by membrane potential, because evidence only suggests the pH upregulation of slow anion channels [27]. In this rule, our hypothesis is that ABI1 is a negative regulator of anion efflux [26]. If ABI1 is present, both positive regulators (cytosolic Ca^2+^ and cytosolic pH) are required to activate the anion efflux; if ABI1 is not present, only one positive regulator is required to activate the anion efflux.

**Depolar^*^ = KEV or AnionEM or (not H^+^ ATPase) or (not KOUT) or Ca^2+^_c_**

Generally speaking, the efflux of positive/negative ions out of the cytosol will hyperpolarize/depolarize the cell. We model K^+^ release from the vacuole to the cytosol and anion efflux and calcium influx across the plasma membrane as positive regulators of depolarization, and plasma membrane H^+^ ATPase and outwardly rectifying K^+^ efflux at the membrane as negative regulators of the depolarization process.

**CaIM^*^ = (ROS or not ERA1 or not ABH1) and (not Depolar)**

Calcium influx across the plasma membrane requires both hyperpolarization and ROS production [28]. CaIM was hypersensitive to ABA in the *era1* mutant, but the hyperpolarization-induced activation threshold of CaIM showed little change [29]. The *abh1* mutant has a similar phenotype to *era1*, except only hypersensitivity in ABA induced Ca^2+^_c_ was reported. Here we hypothesize that ABH1 is regulating CaIM; we evaluated the possibility of ABH1 regulating CIS instead, and the results were similar.

**KOUT^*^ = (pH_c_ or not ROS or not NO) and Depolar**

Membrane depolarization will drive K^+^ flow out of the cell, and the activity of outwardly rectifying K^+^ currents is activated by cytosolic pH increase [30] and inhibited by ROS [31] and nitric oxide [32].

**KAP^*^= (not pH_c_ or not Ca^2+^_c_) and Depolar**

The K^+^ AP current is a transient K^+^ current which was observed in Arabidopsis guard cells only [33]. The characteristic difference in the regulation of KAP is that, unlike KOUT, KAP is inhibited by both pH and cytosolic calcium. The activation of KAP also requires depolarization of the membrane potential.

**KEV^*^ = Ca^2+^_c_**

Calcium-induced K^+^ release through K^+^-permeable channels in the tonoplast [34].

**PEPC^*^ = not ABA**

**Malate^*^ = PEPC and not ABA and not AnionEM**

ABA inhibits PEPC activity in guard cells [35] and PEPC is an upstream enzyme of malate biosynthesis. ABA also regulates malate concentration by inducing malate breakdown [36]. Anion efflux negatively regulates intracellular malate concentration by releasing malate from the cytosol [37].

**RAC1^*^ = not ABA and not ABI1**

**Actin^*^ = Ca^2+^_c_ or not RAC1**

Actin cytoskeleton reorganization has been shown to be a positive regulator of ABA induced stomatal closure, and dominant negative RAC1 expression causes actin cytoskeleton reorganization and stomatal closure [38]. ABI1 negatively regulates ABA inhibition of RAC1 [38].

**Closure^*^ = (KOUT or KAP ) and AnionEM and Actin and not Malate**

Stomatal closure requires both anion efflux from membrane [39] and potassium efflux via either slow outwardly rectifying K^+^ channels [40] or fast transient (AP) K^+^ channels [33]. Malate is a negative regulator of stomatal closure through its effect of osmotic regulation [36], and closure also requires the reorganization of the actin cytoskeleton [38]. The “or” function indicates that either K^+^ current type is strong enough to drive closure; the “and” functions indicate that anion efflux, K^+^ efflux, actin reorganization and malate breakdown are all required to drive efficient stomatal closure.

**References**

1. Coursol S, Fan LM, Le Stunff H, Spiegel S, Gilroy S, et al. (2003) Sphingolipid signalling in *Arabidopsis* guard cells involves heterotrimeric G proteins. Nature 423: 651-654.

2. Jones AM, Assmann SM (2004) Plants: The latest model system for G-protein research. EMBO Rep 5: 572-578.

3. Pandey S, Assmann SM (2004) The Arabidopsis putative G protein-coupled receptor GCR1 interacts with the G protein α subunit GPA1 and regulates abscisic acid signaling. Plant Cell 16: 1616-1632.

4. Zhao J, Wang X (2004) Arabidopsis phospholipase Dα1 interacts with the heterotrimeric G-protein α-subunit through a motif analogous to the DRY motif in G-protein-coupled receptors. J Biol Chem 279: 1794-1800.

5. Suhita D, Raghavendra AS, Kwak JM, Vavasseur A (2004) Cytoplasmic alkalization precedes reactive oxygen species production during methyl jasmonate- and abscisic acid-induced stomatal closure. Plant Physiol 134: 1536-1545.

6. Irving HR, Gehring CA, Parish RW (1992) Changes in cytosolic pH and calcium of guard cells precede stomatal movements. Proc Natl Acad Sci U S A 89: 1790-1794.

7. Mustilli AC, Merlot S, Vavasseur A, Fenzi F, Giraudat J (2002) Arabidopsis OST1 protein kinase mediates the regulation of stomatal aperture by abscisic acid and acts upstream of reactive oxygen species production. Plant Cell 14: 3089-3099.

8. Park J, Gu Y, Lee Y, Yang Z (2004) Phosphatidic acid induces leaf cell death in Arabidopsis by activating the Rho-related small G protein GTPase-mediated pathway of reactive oxygen species generation. Plant Physiol 134: 129-136.

9. Murata Y, Pei ZM, Mori IC, Schroeder J (2001) Abscisic acid activation of plasma membrane Ca^2+^ channels in guard cells requires cytosolic NAD(P)H and is differentially disrupted upstream and downstream of reactive oxygen species production in *abi1-1* and *abi2-1* protein phosphatase 2C mutants. Plant Cell 13: 2513-2523.

10. Zhang X, Wang H, Takemiya A, Song CP, Kinoshita T, et al. (2004) Inhibition of blue light-dependent H^+^ pumping by abscisic acid through hydrogen peroxide-induced

dephosphorylation of the plasma membrane

H^+^ -atpase in guard cell protoplasts. Plant Physiol 136: 4150-4158.

11. Kinoshita T, Nishimura M, Shimazaki K (1995) Cytosolic concentration of Ca^2+^ regulates the plasma membrane H^+^ -ATPase in guard cells of fava bean. Plant Cell 7: 1333-1342.

12. Serrano R (1989) Structure and function of plasma membrane atpase. Annu Rev of Plant Physiol Plant Mol Biol 40: 61-94.

13. Leube MP, Grill E, Amrhein N (1998) ABI1 of Arabidopsis is a protein serine/threonine phosphatase highly regulated by the proton and magnesium ion concentration. FEBS Lett 424: 100-104.

14. Meinhard M, Grill E (2001) Hydrogen peroxide is a regulator of ABI1, a protein phosphatase 2C from Arabidopsis. FEBS Lett 508: 443-446.

15. Zhang W, Qin C, Zhao J, Wang X (2004) Phospholipase Dα1-derived phosphatidic acid interacts with ABI1 phosphatase 2C and regulates abscisic acid signaling. Proc Natl Acad Sci U S A 101: 9508-9513.

16. Guo FQ, Okamoto M, Crawford NM (2003) Identification of a plant nitric oxide synthase gene involved in hormonal signaling. Science 302: 100-103.

17. Desikan R, Griffiths R, Hancock J, Neill S (2002) A new role for an old enzyme: Nitrate reductase-mediated nitric oxide generation is required for abscisic acid-induced stomatal closure in *Arabidopsis thaliana*. Proc Natl Acad Sci U S A 99: 16314-16318.

18. Garcia-Mata C, Gay R, Sokolovski S, Hills A, Lamattina L, et al. (2003) Nitric oxide regulates K^+^ and Cl^-^ channels in guard cells through a subset of abscisic acid-evoked signaling pathways. Proc Natl Acad Sci U S A 100: 11116-11121.

19. Staxen II, Pical C, Montgomery LT, Gray JE, Hetherington AM, et al. (1999) Abscisic acid induces oscillations in guard-cell cytosolic free calcium that involve phosphoinositide-specific phospholipase C. Proc Natl Acad Sci U S A 96: 1779-1784.

20. Lemtiri-Chlieh F, MacRobbie EA, Webb AA, Manison NF, Brownlee C, et al. (2003) Inositol hexakisphosphate mobilizes an endomembrane store of calcium in guard cells. Proc Natl Acad Sci U S A 100: 10091-10095.

21. Otterhag L, Sommarin M, Pical C (2001) N-terminal EF-hand-like domain is required for phosphoinositide-specific phospholipase C activity in Arabidopsis thaliana. FEBS Lett 497: 165-170.

22. Xia HJ, Yang G (2005) Inositol 1,4,5-trisphosphate 3-kinases: Functions and regulations. Cell Res 15: 83-91.

23. Peiter E, Maathuis FJ, Mills LN, Knight H, Pelloux J, et al. (2005) The vacuolar Ca^2+^-activated channel TPC1 regulates germination and stomatal movement. Nature 434: 404-408.

24. Allen GJ, Kwak JM, Chu SP, Llopis J, Tsien RY, et al. (1999) Cameleon calcium indicator reports cytoplasmic calcium dynamics in Arabidopsis guard cells. Plant J 19: 735-747.

25. Allen GJ, Chu SP, Harrington CL, Schumacher K, Hoffmann T, et al. (2001) A defined range of guard cell calcium oscillation parameters encodes stomatal movements. Nature 411: 1053-1057.

26. Allen GJ, Kuchitsu K, Chu SP, Murata Y, Schroeder JI (1999) Arabidopsis *abi1-1* and *abi2-1* phosphatase mutations reduce abscisic acid-induced cytoplasmic calcium rises in guard cells. Plant Cell 11: 1785-1798.

27. Wang XQ, Ullah H, Jones AM, Assmann SM (2001) G protein regulation of ion channels and abscisic acid signaling in *Arabidopsis* guard cells. Science 292: 2070-2072.

28. Pei ZM, Murata Y, Benning G, Thomine S, Klüsener B, et al. (2000) Calcium channels activated by hydrogen peroxide mediate abscisic acid signalling in guard cells. Nature 406: 731-734.

29. Allen GJ, Murata Y, Chu SP, Nafisi M, Schroeder JI (2002) Hypersensitivity of abscisic acid-induced cytosolic calcium increases in the *Arabidopsis* farnesyltransferase mutant *era1-2*. Plant Cell 14: 1649-1662.

30. Miedema H, Assmann SM (1996) A membrane-delimited effect of internal pH on the K^+^ outward rectifier of Vicia faba guard cells. J Membr Biol 154: 227-237.

31. Kohler B, Hills A, Blatt MR (2003) Control of guard cell ion channels by hydrogen peroxide and abscisic acid indicates their action through alternate signaling pathways. Plant Physiol 131: 385-388.

32. Sokolovski S, Blatt MR (2004) Nitric oxide block of outward-rectifying K^+^ channels indicates direct control by protein nitrosylation in guard cells. Plant Physiol 136: 4275-4284.

33. Pei ZM, Baizabal-Aguirre VM, Allen GJ, Schroeder JI (1998) A transient outward-rectifying K^+^ channel current down-regulated by cytosolic Ca^2+^ in *Arabidopsis thaliana* guard cells. Proc Natl Acad Sci U S A 95: 6548-6553.

34. Ward JM, Schroeder JI (1994) Calcium-activated K^+^ channels and calcium-induced calcium release by slow vacuolar ion channels in guard cell vacuoles implicated in the control of stomatal closure. Plant Cell 6: 669-683.

35. Du Z, Aghoram K, Outlaw WH, Jr. (1997) *In vivo* phosphorylation of phospho*enol*pyruvate carboxylase in guard cells of *Vicia faba* l. is enhanced by fusicoccin and suppressed by abscisic acid. Arch Biochem Biophys 337: 345-350.

36. Dittrich P, Raschke K (1977) Malate metabolism in isolated epidermis of *Commelina communis* l. In relation to stomatal functioning. Planta 134: 77-81.

37. Schmidt C, Schroeder JI (1994) Anion selectivity of slow anion channels in the plasma membrane of guard cells (large nitrate permeability). Plant Physiol 106: 383-391.

38. Lemichez E, Wu Y, Sanchez JP, Mettouchi A, Mathur J, et al. (2001) Inactivation of AtRac1 by abscisic acid is essential for stomatal closure. Genes Dev 15: 1808-1816.

39. Schwartz A, Ilan N, Schwarz M, Scheaffer J, Assmann SM, et al. (1995) Anion-channel blockers inhibit S-type anion channels and abscisic acid responses in guard cells. Plant Physiol 109: 651-658.

40. Hosy E, Vavasseur A, Mouline K, Dreyer I, Gaymard F, et al. (2003) The Arabidopsis outward K^+^ channel GORK is involved in regulation of stomatal movements and plant transpiration. Proc Natl Acad Sci U S A 100: 5549-5554.
