# Supplementary material for: Predicting Essential Components of Signal Transduction Networks: A Dynamic Model of Guard Cell Abscisic Acid Signaling
Source: PLoS Biol. 2006 Sep 12;4(10):e312. doi: 10.1371/journal.pbio.0040312 (PMC1564158; doi:10.1371/journal.pbio.0040312)
Supplement: Supplementary file 3 [file pbio.0040312.sd002.doc]

**Text S2: Verification of the Inference Process and of the Resulting Network**

Table S1 is based on human curation of published data, and consequently displays specific history-dependent features. For example, the earlier one component/process was published, the more frequently it will appear as a part of an entry in Table S1. The most frequently recorded result is “ABA induces closure” (35 times), all in the “process B” column; this is simply because “ABA induces closure” is a well-known fact and was tested in many experiments as a control to study other components in the process. Another example is that pH_c_ appears in 7 entries but Ca^2+^_c_ appears in 29 entries, probably because the technique used in measuring intracellular Ca^2+^ is implemented in more laboratories than the technique used in measuring intracellular pH. To gauge the stability of Figure 2 (in the main text) with respect to the addition or removal of information from Table S1, we calculated the minimum number of entries involving a given node that need to be removed before we lose a path from ABA to closure mediated by that node. The table below indicates the total number of entries in Table S1 for network components predicted by our model to be important, and shows the minimum number of entries whose deletion is necessary to cause the loss of the ABA-closure path containing the component.

| Node | Minimum number of deleted entries | Total number of related entries | Ratio of previous two columns |
| --- | --- | --- | --- |
| KOUT | 5 | 10 | 0.5 |
| Ca^2+^_c_ | 4 | 29 | 0.14 |
| ROS | 4 | 23 | 0.17 |
| Depolar | 4 | 13 | 0.30 |
| PLD | 4 | 6 | 0.66 |
| pH_c_ | 4 | 7 | 0.57 |
| AnionEM | 3 | 19 | 0.16 |
| S1P | 3 | 4 | 0.75 |
| PA | 3 | 7 | 0.42 |
| ABI1 | 3 | 7 | 0.42 |
| SphK | 1 | 4 | 0.25 |
| GPA1 | 1 | 4 | 0.25 |
| Actin | 1 | 3 | 0.33 |

The table reveals a correlation between the number of interactions/relationships known about a node and the difficulty of removing a path from ABA to closure passing through that node by the elimination of table entries. The ratio between the minimum required information and the existing information does not seem biased, however.
