# Supplementary material for: Predicting Essential Components of Signal Transduction Networks: A Dynamic Model of Guard Cell Abscisic Acid Signaling
Source: PLoS Biol. 2006 Sep 12;4(10):e312. doi: 10.1371/journal.pbio.0040312 (PMC1564158; doi:10.1371/journal.pbio.0040312)
Supplement: Supplementary file 4 [file pbio.0040312.sd003.doc]

**Text S3: The Effect of Random Rewiring on the Network Dynamics**

To gauge the uniqueness of our reconstructed network in reproducing ABA-induced closure, we test its dynamics’ resilience to random edge rewiring. As the interchange of a positive regulation with a negative regulation would change the sign of two paths in the model, which will definitely change the dynamics, we randomize the network in a more restricted way. We follow the rewiring methods used in [1] and we further preserve not only the degree but also the sign of the edges in the model. We gradually increase the degree of randomization from one pair of edges up to eight pairs of edges. After network randomization we test whether such a model can still show ABA-induced closure following the dynamic simulation methods described in the main text. We classify the effect of ABA-induced closure in the simulation after the probability of closure reaches a stationary value. If this value is 1 (100%), we name it “reaches unity”; this category corresponds to the normal, hyposensitive and hypersensitive responses we described in the main text. If the value is 0% we name it “reaches zero”, this category corresponding to the insensitive responses in the model. And if the stationary probability of closure is between 0 and 1, we name it “reduced”. Note that in this analysis we use only three of the five categories described in the main text because two of those categories (hypersensitivity and hyposensitivity) characterize the effect of disruption of nodes as compared to the normal response. We did 400 random rewirings for positive edge pairs, and 100 random rewirings for negative pairs, because there are twice as many positive edges in our model as negative edges. We summarize the results in the two bar graphs shown in Figure S1. As expected from the redundancy of the network, when only one pair of either positive edges or negative edges is randomized, in more than 70% of randomized models we still see ABA-induced closure (yellow bars). As the degree of randomization increases, the number of randomized networks that can produce ABA-induction of closure is reduced, whereas the number of reduced/insensitive networks increases. With randomized negative pairs, networks where ABA induces no closure (maroon bars) are more likely, in agreement with intuitive expectation.

Figure S1: Probability of closure in randomized networks where pairs of positive (A) or negative (B) edges are rewired. The x axis indicates the number of rewired edge pairs, from zero (control) up to eight. The bar graphs display the distribution of dynamic behaviors in a large number of randomized networks (the sum of the three segments is 100%). Yellow denotes response that reaches unity (100% closure), blue indicates reduced response (less than 100% closure), and maroon means response that reaches zero (0% closure).

**References**

1. Shen-Orr SS, Milo R, Mangan S, Alon U (2002) Network motifs in the transcriptional regulation network of escherichia coli. Nat Genet 31: 64-68.
