# Supplementary material for: Predicting Essential Components of Signal Transduction Networks: A Dynamic Model of Guard Cell Abscisic Acid Signaling
Source: PLoS Biol. 2006 Sep 12;4(10):e312. doi: 10.1371/journal.pbio.0040312 (PMC1564158; doi:10.1371/journal.pbio.0040312)
Supplement: Supplementary file 5 [file pbio.0040312.st001.doc]

**Table S1**. **Synthesis of Experimental Information about Regulatory Interactions between ABA Signal Transduction Pathway Components.**

The explanation of the abbreviations used in the first two columns can be found in the caption of Figure 2 (in the main text). Each entry in the database consists of a source node (Node A), a target node or target process (Node/Process B), their interaction, the plant species this interaction was observed in, and references. In the column “Node/Process B,” → means activation, ---| means inhibition; both can be enzymatic processes, e.g. protein phosphorylation, protein dephosphorylation etc. In the interaction column, five different interactions are allowed: “promotes”, “partially promotes”, “inhibits”, “partially inhibits”, and “no relation”. “A promotes B” is inferred if in the absence of A, B cannot be observed; “A inhibits B” is inferred if in the absence of A, B is upregulated. A promotes/inhibits process B means that in the absence of A, process B

cannot happen/is upregulated. “Partially promotes” or “partially inhibits” means that B is not completely controlled by A. “No relation” means that the node or process B is not affected by changes in A. The reference list gives representative references; in some cases the phenomenon was described in multiple reports, and additional literature references are given in the main text.

| Node A | Node/Process B | Interaction | Species | References |
| --- | --- | --- | --- | --- |
| ABA | SphK | Promotes | *Arabidopsis* | *[1]* |
| ABA | AAPK (OST1 homologue) | Promotes | *Vicia faba* | *[2]* |
| ABA | CaIM | Promotes | *Vicia faba* | *[3]* |
| ABA | NO | Promotes | *Arabidopsis* | *[4]* |
| ABA | Ca^2+^_c_ | Promotes | *Commelina communis* | *[5]* |
| ABA | PLD → PA | Promotes | *Vicia faba* | *[6]* |
| ABA | PLD | Promotes | *Vicia faba* | *[6]* |
| ABA | H^+^ ATPase | Partially inhibits | *Vicia faba* | *[7,8]* |
| ABA | RAC1 (ROP6) | Inhibits | *Arabidopsis* | *[9]* |
| ABA | ADPRc | Promotes | *Arabidopsis* | *[10]* |
| ABA | Ca^2+^_c_ oscillation | Promotes | *Arabidopsis* | *[11]* |
| ABA | InsP3 | Promotes | *Vicia faba* | *[8]* |
| ABA | InsP6 | Promotes | *S. tuberosum* | *[12]* |
| ABA | OST1 | Promotes | *Arabidopsis* | *[13]* |
| ABA | ROS | Partially promotes | *Vicia faba* | *[14]* |
| ABA | Hyperpolarization → Ca^2+^_c_ | Promotes | *Vicia faba* | *[15]* |
| ABA | PEPC | Inhibits | *Vicia faba* | *[16]* |
| ABA | Malate | Partially inhibits | *Commelina communis* | *[17]* |
| SphK | ABA→ Closure | Partially promotes | *Arabidopsis* | *[1]* |
| SphK | ABA → AnionEM | Partially promotes | *Arabidopsis* | *[1]* |
| SphK | ABA → S1P | Promotes | *Arabidopsis* | *[1]* |
| S1P | ABA → Closure | Promotes | *Arabidopsis* | *[1]* |
| GPA1 | S1P → AnionEM | Promotes | *Arabidopsis* | *[1]* |
| GPA1 | ABA → ROS | Promotes | *Arabidopsis* | *Unpublished* |
| GPA1 | ABA → AnionEM | Promotes | *Arabidopsis* | *[18]* |
| GPA1 | PLD | Promotes | *Arabidopsis* | *[19]* |
| GCR1 | ABA → Closure | Inhibits | *Arabidopsis* | *[20]* |
| GCR1 | ABA → Closure | Partially inhibits | *Arabidopsis* | *[20]* |
| GCR1 | S1P → Closure | Partially inhibits | *Arabidopsis* | *[20]* |
| PLD | Phospholipids → PA | Promotes | *Vicia faba* | *[6]* |
| PLD | ABA → Closure | Promotes | *Vicia faba* | *[6]* |
| PA | ROS | Promotes | *Arabidopsis* | *[21]* |
| PA | ABI1 | Inhibits | *Arabidopsis* | *[22]* |
| ROP2 | PA → ROS | Partially promotes | *Arabidopsis* | *[21]* |
| RCN1 | ABA → Closure | Promotes | *Arabidopsis* | *[23]* |
| RCN1 | ABA → AnionEM | Promotes | *Arabidopsis* | *[23]* |
| RCN1 | ABA → Ca^2+^_c_ | Promotes | *Arabidopsis* | *[23]* |
| OST1 | ABA → Closure | Promotes | *Arabidopsis* | *[13]* |
| OST1 | ABA → ROS | Promotes | *Arabidopsis* | *[13]* |
| OST1 | ROS → Closure | No relation | *Arabidopsis* | *[13]* |
| PLC | ABA → Ca^2+^_c_ | Promotes | *Arabidopsis* | *[24]* |
| PLC | ABA → Closure | Partially promotes | *Vicia faba* | *[6]* |
| PLC | ABA → Closure | Promotes | *Commelina communis* | *[25,26]* |
| PLC | ABA 🡪 Ca^2+^_c_ | Partially  Promotes | *Commelina communis* | *[26]* |
| PLC | ABA → Closure | Partially promotes | *Nicotiana rustica* | *[27]* |
| PLC | ABA → KOUT | Partially promotes | *Commelina communis* | *[28]* |
| RAC1 (ROP6) | Actin disruption | Partially inhibits | *Arabidopsis* | *[9]* |
| RAC1 (ROP6) | Closure | Partially inhibits | *Arabidopsis* | *[9]* |
| RAC1 (ROP6) | ABA → Closure | Partially inhibits | *Arabidopsis* | *[9]* |
| NIA12 | ABA → Closure | Promotes | *Arabidopsis* | *[4]* |
| NIA12 | Nitrite → Closure | Promotes | *Arabidopsis* | *[4]* |
| NIA12 | ABA → NO | Promotes | *Arabidopsis* | *[4]* |
| NIA12 | Nitrite → NO | Promotes | *Arabidopsis* | *[4]* |
| NIA12 | NO → Closure | No relation | *Arabidopsis* | *[4]* |
| NIA12 | ROS → Closure | No relation | *Arabidopsis* | *[4]* |
| Nitrite | NIA12 → NO | Substrate | *Arabidopsis* | *[4]* |
| NOS | ABA → Closure | Promotes | *Arabidopsis* | *[29]* |
| NOS | ABA → NO | Promotes | *Arabidopsis* | *[29]* |
| NO | ABA → Closure | Promotes | *Pisum sativum* | *[30]* |
| NO | ABA → Closure | Promotes | *Arabidopsis* | *[4]* |
| NO | AnionEM | Promotes | *Vicia faba* | *[31]* |
| NO | ABA → AnionEM | Promotes | *Vicia faba* | *[31]* |
| NO | KOUT | Partially inhibits | *Arabidopsis* | *[32]* |
| NO | Ca^2+^_c_→ CIS | Partially promotes | *Vicia faba* | *[31]* |
| NO | Hyperpolarization → CaIM | Inhibits | *Vicia faba* | *[31]* |
| ADPRc | NO → Ca^2+^_c_ | Promotes | *Vicia faba* | *[31]* |
| cADPR | Ca^2+^ _c_→ CIS | Promotes | *Vicia faba* | *[31]* |
| cADPR | ABA → KOUT | Partially promotes | *Commelina communis* | *[28]* |
| GC | NO → Ca^2+^_c_ | Promotes | *Vicia faba* | *[31]* |
| InsPK | InsP6 | Promotes | *S. tuberosum* | *[12]* |
| InsP6 | CIS | Partially promotes | *Vicia faba* | *[33]* |
| pH_c_ | ABA → AnionEM | Promotes | *Arabidopsis* | *[18]* |
| pH_c_ | ABA → Closure | Promotes | *Arabidopsis* | *[18]* |
| pH_c_ | ROS | Partially promotes | *Arabidopsis* | *[34]* |
| pH_c_ | KOUT | Promotes | *Vicia faba* | *[35]* |
| pH_c_ | H^+^ ATPase | Inhibits | *Vicia faba* | *[36]* |
| pH_c_ | Depolarization → KAP | Inhibits | *Arabidopsis* | *[37]* |
| pH_c_ | ABI1 | Promotes | *Arabidopsis* | *[38]* |
| Atrboh | ABA → CaIM | Promotes | *Arabidopsis* | *[39]* |
| Atrboh | ROS → CaIM | No relation | *Arabidopsis* | *[39]* |
| NADPH | ABA → CalM | Promotes | *Arabidopsis* | *[40]* |
| NADH | ABA → CalM | Partially promotes | *Arabidopsis* | *[40]* |
| ROS | ABA → Closure | Partially promotes | *Vicia faba* | *[14]* |
| ROS | Closure | Partially promotes | *Vicia faba* | *[14]* |
| ROS | ABA → CaIM | Promotes | *Vicia faba* | *[41]* |
| ROS | KOUT | Inhibits | *Vicia faba* | *[41]* |
| ROS | ABA ---\| H^+^ ATPase | Partially promotes | *Vicia faba* | *[42]* |
| ROS | ABI1 | Inhibits | *Arabidopsis* | *[43]* |
| ERA1 | ABA → CalM | Inhibits | *Arabidopsis* | *[44]* |
| ERA1 | ABA → Closure | Inhibits | *Arabidopsis* | *[44]* |
| ERA1 | ABA → CaIM | Inhibits | *Arabidopsis* | *[44]* |
| ERA1 | Ca^2+^_c_ → AnionEM | No relation | *Arabidopsis* | *[44]* |
| ROP10 | ABA → Closure | Partially inhibits | *Arabidopsis* | *[45]* |
| ERA1 | ROP10 | Partially promotes | *Arabidopsis* | *[45]* |
| ABH1 | ABA 🡪 Ca^2+^_c_ | Partially inhibits | *Arabidopsis* | *[46]* |
| ABH1 | ABA 🡪 Closure | Partially inhibits | *Arabidopsis* | *[46]* |
| ABH1 | AnionEM | Partially inhibits | *Arabidopsis* | *[47]* |
| CaIM | ABA → KOUT | Partially promotes | *Commelina communis* | *[28]* |
| Ca^2+^_c_ | ABA → OST1 | No relation | *Arabidopsis* | *[13]* |
| Ca^2+^_c_ | Depolarization → KAP | Partially inhibits | *Arabidopsis* | *[37]* |
| Ca^2+^_c_ | H^+^ ATPase | Inhibits | *Vicia faba* | *[48]* |
| Ca^2+^_c_ | PLC 🡪 InsP3 | Promotes | *Arabidospis* | *[49]* |
| Ca^2+^_c_ | AnionEM | Promotes | *Arabidopsis* | *[11]* |
| Ca^2+^_c_ | InsP6 → CIS | No relation | *Vicia faba* | *[33]* |
| Ca^2+^_c_ | KOUT | no relation | *S. tuberosum* | *[12]* |
| Ca^2+^_c_ | ABA 🡪 Actin disruption | Promotes | *Commelina communis* | *[50]* |
| Ca^2+^_c_ | NO → AnionEM | Promotes | *Vicia faba* | *[31]* |
| Ca^2+^_c_ | KEV | Promotes | *Commelina communis* | *[28]* |
| ABI1 | ABA --\| RAC1 (ROP6) | Partially | *Arabidopsis* | *[9]* |
| ABI1 | ABA 🡪 AnionEM | Inhibits | *Arabidopsis* | *[11,51]* |
| ABI1 | ABA 🡪 ROS | Inhibits | *Arabidopsis* | *[11,40]* |
| ABI1 | ABA🡪 Ca^2+^_c_ | Inhibits | *Arabidopsis* | *[11]* |
| Depolarization | KOUT | Promotes | *Arabidopsis* | *[52]* |
| Hyperpolarization | Depolarization → KAP | Promotes | *Arabidopsis* | *[37]* |
| Hyperpolarization | Ca^2+^_c_ | Promotes | *Vicia faba* | *[15]* |
| Hyperpolarization | Ca^2+^_c_ dynamics | No relation | *Vicia faba* | *[15]* |
| KOUT | ABA → Closure | Promotes | *Arabidopsis* | *[52]* |
| AnionEM | ABA → Closure | Promotes | *Arabidopsis* | *[53]* |
| AnionEM | ABA → Closure | Partially promotes | *Commelina communis* | *[54]* |
| AtP2C-HA | ABA 🡪 Closure | Partially inhibits | *Arabidopsis* | *[55]* |
| AtPP2CA | ABA 🡪 Closure | Partially inhibits | *Arabidopsis* | *[56]* |

**References**

1. Coursol S, Fan LM, Le Stunff H, Spiegel S, Gilroy S, et al. (2003) Sphingolipid signalling in *Arabidopsis* guard cells involves heterotrimeric G proteins. Nature 423: 651-654.

2. Li J, Assmann SM (1996) An abscisic acid-activated and calcium-independent protein kinase from guard cells of fava bean. Plant Cell 8: 2359-2368.

3. Hamilton DW, Hills A, Kohler B, Blatt MR (2000) Ca^2+^ channels at the plasma membrane of stomatal guard cells are activated by hyperpolarization and abscisic acid. Proc Natl Acad Sci U S A 97: 4967-4972.

4. Desikan R, Griffiths R, Hancock J, Neill S (2002) A new role for an old enzyme: Nitrate reductase-mediated nitric oxide generation is required for abscisic acid-induced stomatal closure in *Arabidopsis thaliana*. Proc Natl Acad Sci U S A 99: 16314-16318.

5. Webb AA, Larman MG, Montgomery LT, Taylor JE, Hetherington AM (2001) The role of calcium in ABA-induced gene expression and stomatal movements. Plant J 26: 351-362.

6. Jacob T, Ritchie S, Assmann SM, Gilroy S (1999) Abscisic acid signal transduction in guard cells is mediated by phospholipase D activity. Proc Natl Acad Sci U S A 96: 12192-12197.

7. Goh CH, Kinoshita T, Oku T, Shimazaki K (1996) Inhibition of blue light-dependent H^+^ pumping by abscisic acid in *Vicia* guard-cell protoplasts. Plant Physiol 111: 433-440.

8. Lee Y, Choi YB, Suh S, Lee J, Assmann SM, et al. (1996) Abscisic acid-induced phosphoinositide turnover in guard cell protoplasts of *Vicia faba*. Plant Physiol 110: 987-996.

9. Lemichez E, Wu Y, Sanchez JP, Mettouchi A, Mathur J, et al. (2001) Inactivation of AtRac1 by abscisic acid is essential for stomatal closure. Genes Dev 15: 1808-1816.

10. Sanchez JP, Duque P, Chua NH (2004) ABA activates ADPR cyclase and cADPR induces a subset of ABA-responsive genes in *Arabidopsis*. Plant J 38: 381-395.

11. Allen GJ, Kuchitsu K, Chu SP, Murata Y, Schroeder JI (1999) Arabidopsis *abi1-1* and *abi2-1* phosphatase mutations reduce abscisic acid-induced cytoplasmic calcium rises in guard cells. Plant Cell 11: 1785-1798.

12. Lemtiri-Chlieh F, MacRobbie EA, Brearley CA (2000) Inositol hexakisphosphate is a physiological signal regulating the K^+^-inward rectifying conductance in guard cells. Proc Natl Acad Sci U S A 97: 8687-8692.

13. Mustilli AC, Merlot S, Vavasseur A, Fenzi F, Giraudat J (2002) Arabidopsis OST1 protein kinase mediates the regulation of stomatal aperture by abscisic acid and acts upstream of reactive oxygen species production. Plant Cell 14: 3089-3099.

14. Zhang X, Zhang L, Dong F, Gao J, Galbraith DW, et al. (2001) Hydrogen peroxide is involved in abscisic acid-induced stomatal closure in *Vicia faba*. Plant Physiol 126: 1438-1448.

15. Grabov A, Blatt MR (1998) Membrane voltage initiates Ca^2+^ waves and potentiates Ca^2+^ increases with abscisic acid in stomatal guard cells. Proc Natl Acad Sci U S A 95: 4778-4783.

16. Du Z, Aghoram K, Outlaw WH, Jr. (1997) *In vivo* phosphorylation of phospho*enol*pyruvate carboxylase in guard cells of *Vicia faba* l. Is enhanced by fusicoccin and suppressed by abscisic acid. Arch Biochem Biophys 337: 345-350.

17. Dittrich P, Raschke K (1977) Malate metabolism in isolated epidermis of *Commelina communis* l. In relation to stomatal functioning. Planta 134: 77-81.

18. Wang XQ, Ullah H, Jones AM, Assmann SM (2001) G protein regulation of ion channels and abscisic acid signaling in *Arabidopsis* guard cells. Science 292: 2070-2072.

19. Zhao J, Wang X (2004) Arabidopsis phospholipase Dα1 interacts with the heterotrimeric G-protein α-subunit through a motif analogous to the DRY motif in G-protein-coupled receptors. J Biol Chem 279: 1794-1800.

20. Pandey S, Assmann SM (2004) The Arabidopsis putative G protein-coupled receptor GCR1 interacts with the G protein α subunit GPA1 and regulates abscisic acid signaling. Plant Cell 16: 1616-1632.

21. Park J, Gu Y, Lee Y, Yang Z (2004) Phosphatidic acid induces leaf cell death in Arabidopsis by activating the Rho-related small G protein GTPase-mediated pathway of reactive oxygen species generation. Plant Physiol 134: 129-136.

22. Zhang W, Qin C, Zhao J, Wang X (2004) Phospholipase Dα1-derived phosphatidic acid interacts with ABI1 phosphatase 2C and regulates abscisic acid signaling. Proc Natl Acad Sci U S A 101: 9508-9513.

23. Kwak JM, Moon JH, Murata Y, Kuchitsu K, Leonhardt N, et al. (2002) Disruption of a guard cell-expressed protein phosphatase 2A regulatory subunit, *RCN1*, confers abscisic acid insensitivity in Arabidopsis. Plant Cell 14: 2849-2861.

24. Klüsener B, Young JJ, Murata Y, Allen GJ, Mori IC, et al. (2002) Convergence of calcium signaling pathways of pathogenic elicitors and abscisic acid in Arabidopsis guard cells. Plant Physiol 130: 2152-2163.

25. Mills LN, Hunt L, Leckie CP, Aitken FL, Wentworth M, et al. (2004) The effects of manipulating phospholipase C on guard cell ABA-signalling. J Exp Bot 55: 199-204.

26. Staxen II, Pical C, Montgomery LT, Gray JE, Hetherington AM, et al. (1999) Abscisic acid induces oscillations in guard-cell cytosolic free calcium that involve phosphoinositide-specific phospholipase C. Proc Natl Acad Sci U S A 96: 1779-1784.

27. Hunt L, Mills LN, Pical C, Leckie CP, Aitken FL, et al. (2003) Phospholipase C is required for the control of stomatal aperture by ABA. Plant J 34: 47-55.

28. MacRobbie EA (2000) ABA activates multiple Ca^2+^ fluxes in stomatal guard cells, triggering vacuolar K^+^(Rb^+^) release. Proc Natl Acad Sci U S A 97: 12361-12368.

29. Guo FQ, Okamoto M, Crawford NM (2003) Identification of a plant nitric oxide synthase gene involved in hormonal signaling. Science 302: 100-103.

30. Neill SJ, Desikan R, Clarke A, Hancock JT (2002) Nitric oxide is a novel component of abscisic acid signaling in stomatal guard cells. Plant Physiol 128: 13-16.

31. Garcia-Mata C, Gay R, Sokolovski S, Hills A, Lamattina L, et al. (2003) Nitric oxide regulates K^+^ and Cl^-^ channels in guard cells through a subset of abscisic acid-evoked signaling pathways. Proc Natl Acad Sci U S A 100: 11116-11121.

32. Sokolovski S, Blatt MR (2004) Nitric oxide block of outward-rectifying K^+^ channels indicates direct control by protein nitrosylation in guard cells. Plant Physiol 136: 4275-4284.

33. Lemtiri-Chlieh F, MacRobbie EA, Webb AA, Manison NF, Brownlee C, et al. (2003) Inositol hexakisphosphate mobilizes an endomembrane store of calcium in guard cells. Proc Natl Acad Sci U S A 100: 10091-10095.

34. Suhita D, Raghavendra AS, Kwak JM, Vavasseur A (2004) Cytoplasmic alkalization precedes reactive oxygen species production during methyl jasmonate- and abscisic acid-induced stomatal closure. Plant Physiol 134: 1536-1545.

35. Miedema H, Assmann SM (1996) A membrane-delimited effect of internal pH on the K^+^ outward rectifier of Vicia faba guard cells. J Membr Biol 154: 227-237.

36. Serrano R (1993) Structure, function and regulation of plasma membrane H^+^-atpase. FEBS Lett 325: 108-111.

37. Pei ZM, Baizabal-Aguirre VM, Allen GJ, Schroeder JI (1998) A transient outward-rectifying K^+^ channel current down-regulated by cytosolic Ca^2+^ in *Arabidopsis thaliana* guard cells. Proc Natl Acad Sci U S A 95: 6548-6553.

38. Leube MP, Grill E, Amrhein N (1998) ABI1 of Arabidopsis is a protein serine/threonine phosphatase highly regulated by the proton and magnesium ion concentration. FEBS Lett 424: 100-104.

39. Kwak JM, Mori IC, Pei ZM, Leonhardt N, Torres MA, et al. (2003) NADPH oxidase *AtrbohD* and *AtrbohF* genes function in ROS-dependent ABA signaling in *Arabidopsis*. Embo J 22: 2623-2633.

40. Murata Y, Pei ZM, Mori IC, Schroeder J (2001) Abscisic acid activation of plasma membrane Ca^2+^ channels in guard cells requires cytosolic NAD(P)H and is differentially disrupted upstream and downstream of reactive oxygen species production in *abi1-1* and *abi2-1* protein phosphatase 2C mutants. Plant Cell 13: 2513-2523.

41. Kohler B, Hills A, Blatt MR (2003) Control of guard cell ion channels by hydrogen peroxide and abscisic acid indicates their action through alternate signaling pathways. Plant Physiol 131: 385-388.

42. Zhang X, Wang H, Takemiya A, Song CP, Kinoshita T, et al. (2004) Inhibition of blue light-dependent H^+^ pumping by abscisic acid through hydrogen peroxide-induced dephosphorylation of the plasma membrane H^+^ -ATPase in guard cell protoplasts. Plant Physiol 136: 4150-4158.

43. Meinhard M, Grill E (2001) Hydrogen peroxide is a regulator of ABI1, a protein phosphatase 2C from Arabidopsis. FEBS Lett 508: 443-446.

44. Allen GJ, Murata Y, Chu SP, Nafisi M, Schroeder JI (2002) Hypersensitivity of abscisic acid-induced cytosolic calcium increases in the *Arabidopsis* farnesyltransferase mutant *era1-2*. Plant Cell 14: 1649-1662.

45. Zheng ZL, Nafisi M, Tam A, Li H, Crowell DN, et al. (2002) Plasma membrane-associated ROP10 small GTPase is a specific negative regulator of abscisic acid responses in Arabidopsis. Plant Cell 14: 2787-2797.

46. Hugouvieux V, Kwak JM, Schroeder JI (2001) An mRNA cap binding protein, ABH1, modulates early abscisic acid signal transduction in Arabidopsis. Cell 106: 477-487.

47. Hugouvieux V, Murata Y, Young JJ, Kwak JM, Mackesy DZ, et al. (2002) Localization, ion channel regulation, and genetic interactions during abscisic acid signaling of the nuclear mRNA cap-binding protein, ABH1. Plant Physiol 130: 1276-1287.

48. Kinoshita T, Nishimura M, Shimazaki K (1995) Cytosolic concentration of Ca^2+^ regulates the plasma membrane H^+^ -atpase in guard cells of fava bean. Plant Cell 7: 1333-1342.

49. Otterhag L, Sommarin M, Pical C (2001) N-terminal EF-hand-like domain is required for phosphoinositide-specific phospholipase C activity in Arabidopsis thaliana. FEBS Lett 497: 165-170.

50. Hwang JU, Lee Y (2001) Abscisic acid-induced actin reorganization in guard cells of dayflower is mediated by cytosolic calcium levels and by protein kinase and protein phosphatase activities. Plant Physiol 125: 2120-2128.

51. Pei ZM, Kuchitsu K, Ward JM, Schwarz M, Schroeder JI (1997) Differential abscisic acid regulation of guard cell slow anion channels in Arabidopsis wild-type and *abi1* and *abi2* mutants. Plant Cell 9: 409-423.

52. Hosy E, Vavasseur A, Mouline K, Dreyer I, Gaymard F, et al. (2003) The Arabidopsis outward K^+^ channel GORK is involved in regulation of stomatal movements and plant transpiration. Proc Natl Acad Sci U S A 100: 5549-5554.

53. Klein M, Perfus-Barbeoch L, Frelet A, Gaedeke N, Reinhardt D, et al. (2003) The plant multidrug resistance ABC transporter AtMRP5 is involved in guard cell hormonal signaling and water use. Plant J 33: 119-129.

54. Leonhardt N, Vavasseur A, Forestier C (1999) ATP binding cassette modulators control abscisic acid-regulated slow anion channels in guard cells. Plant Cell 11: 1141-1152.

55. Leonhardt N, Kwak JM, Robert N, Waner D, Leonhardt G, et al. (2004) Microarray expression analyses of Arabidopsis guard cells and isolation of a recessive abscisic acid hypersensitive protein phosphatase 2C mutant. Plant Cell 16: 596-615.

56. Kuhn JM, Boisson-Dernier A, Dizon MB, Maktabi MH, Schroeder JI (2006) The protein phosphatase AtPP2CA negatively regulates abscisic acid signal transduction in Arabidopsis, and effects of abh1 on AtPP2CA mRNA. Plant Physiol 140: 127-139.
